# Nowcasting and Forecasting the Monthly Food Stamps Data in the US Using Online Search Data

**DOI:** 10.1371/journal.pone.0111894

**Published:** 2014-11-04

**Authors:** Dean Fantazzini

**Affiliations:** Moscow School of Economics, Moscow State University, Moscow, Russia; University of Warwick, United Kingdom

## Abstract

We propose the use of Google online search data for nowcasting and forecasting the number of food stamps recipients. We perform a large out-of-sample forecasting exercise with almost 3000 competing models with forecast horizons up to 2 years ahead, and we show that models including Google search data statistically outperform the competing models at all considered horizons. These results hold also with several robustness checks, considering alternative keywords, a falsification test, different out-of-samples, directional accuracy and forecasts at the state-level.

## Introduction

**Figure 1:**
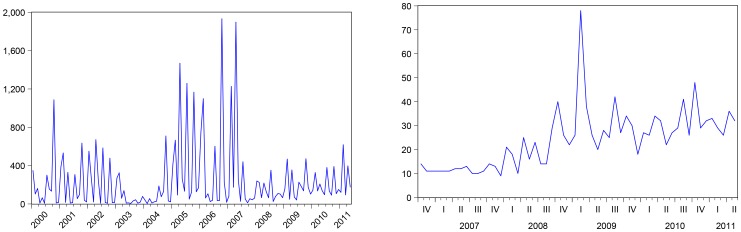
Bloomberg story-count for “food stamps” worldwide (left plot); Google standardized volume of news related to “food stamps” worldwide (right plot). Google data are registered trademarks of Google Inc., used with permission.

The Supplemental Nutrition Assistance Program (SNAP), which was known as the Food Stamp Program until it was renamed in the 2008 US farm bill, is a federal aid program designed to give low- and no-income people living in the US a means to buy food. Since 2011, more than 40 million Americans have received this kind of aid. The number of monthly food stamps recipients has become increasingly scrutinized worldwide as an important indicator of the US economy: see Figure 1 which reports the monthly (absolute) number of news related to food stamps in Bloomberg since 2000, and the monthly (standardized) number of news in Google since 2006 worldwide.

There are several reasons behind this phenomenon: one is the lack of trust in classical indicators like the GDP, particularly during the last global recession, due to subsequent downward GDP revisions. This has sparked a hot debate about the veracity of official data, forcing even an official declaration by Mark Doms, the Chief Economist of the US Department of Commerce, who said on the 26/11/2011 that “…*as many outside economists and GDP experts will attest to, the integrity of BEA [Bureau of Economic Analysis]'s data and its recent revisions to the latest U.S. recessionary period should not be suspect. But there is always room for improvement, and BEA and the Commerce Department continue to search for ways to improve its data collection and analysis to best serve the American people*”(see the full note by Mark Doms at http://www.esa.doc.gov/Blog/2011/08/26/no-smoke-and-mirrors-gdp-note-bea's-recent-revisions). Another reason is the criticism about the official unemployment rate: the official rate is the so-called U3 (i.e. people without jobs who have actively looked for work within the past four weeks) which can be quite restrictive and underestimate the real rate. Many analysts prefer to consider U6 ( = U3 + “discouraged workers” + “marginally attached workers” + Part-time workers who want to work full-time, but cannot due to economic reasons), but even this last measure does not include long-term discouraged workers, which were excluded by the US Bureau of Labor Statistics in 1994. Finally, in 2008, Moody's Analytics found that food stamps were the most effective form of economic stimulus, increasing economic activity by $1.73 for every dollar spent (that is, the one-year fiscal multiplier effect). Unemployment insurance came in second, at $1.62, whereas most tax cuts yielded a dollar or less. The reason for this high effectiveness is the fact that “…*food stamps recipients are so poor that they tend to spend them immediately*”, [1]. In 2011, US Secretary of Agriculture Tom Vilsack gave a higher estimate of $1.84, based on a 2002 USDA study.

Given this background, models for nowcasting (i.e. forecasting in real time, since the official release is published with a 2-month lag) can be very important for financial analysts and economists, since they do not have access to the initial estimates by the USDA, which are not released due to the high noise in the data. Moreover, models for forecasting can be very important for policy makers like the USDA when preparing public budgets: for example, it can be of great interest to know when an increase of the number of food stamps recipients will start abating. Similarly, economists and financial professionals worldwide can benefit from good forecasts, since the number of food stamps recipients is an important indicator of the US economy.

Unfortunately, food stamp caseloads are difficult to predict and the academic literature in this regard is very limited: the main paper dealing with food stamps forecasting is in fact the one by [2] for the USDA in 1991. Despite an extensive modelling effort, [2] concluded that their “[…] *model did not yield highly accurate forecasts of the Food Stamp caseload*”, and that “*none of the […] models would have captured the increase in participation that began in 1989*”. This is probably one of the reason why the (vast) literature since then mainly focused only on the determinants of welfare caseloads, analyzing the effects of SNAP policies, welfare policies, and the economy on SNAP participation rates and other characteristics, without dealing with forecasting: see the recent study by [3], the review by [4] and references therein for a discussion and an overview of this literature. A much smaller strand of the literature kept on dealing with welfare caseload forecasting, even though on a more limited scale than [2] –only at the state level– and not always specifically with the food stamps program: [5] discussed the forecasting of child abuse and neglect reports in urban, suburban, and rural counties; [6] dealt with the income assistance caseloads for the state of Washington; [7] developed a forecasting model for the Aid to Families with Dependent Children (AFDC) caseloads; [8] dealt with monthly state-level welfare caseloads in California; [9] provided a review of the literature about welfare caseloads and forecasting methods at the state level, showing an example with Georgia Temporary Assistance for Needy Families (TANF) caseloads, while [10] forecasted the number of participants in the special supplemental nutrition program for Women, Infants, and Children (WIC) using Vector Autoregression and ARIMA models. Differently from the previous literature, [11] is the first work to employ several methods to forecast Japanese welfare caseloads at the national level and to compare their performances.

Twenty years after [2], many interesting models have been developed: cointegration methods, nonlinear methods, periodic models. Even more interesting, now we have free access to Google online search data. Google holds the world leadership among all search engines with 82.8% market share (Net Applications, 2013) and it receives several hundred million queries each day: since January 2004, Google has started to make available the standardized number of the internet search queries for a keyword (or a group of keywords) with a tool called *Google Trends*. It provides information of users' relative interest for a particular search query at a given geographic region and at a given time. The Google Index (GI) for a specific query is standardized between 0 to 100%, where 100% is the peak of the search queries. The academic literature has started using Google search data for both forecasting and nowcasting purposes: [12] proposed Google Trends data for predicting various economic and financial indicators [13], used Google search data for forecasting the German unemployment rate [14], for the Italian unemployment rate [15], for the Israeli unemployment rate, while [16] for the US unemployment rate [17], [18] and [19]. estimated the ‘influenza’ activity in the US, China and South Korea, respectively, using online influenza-related internet queries [20]. used Google data to measure investors' attention for a sample of Russell 3000 stocks, while [21] used Google data to forecast the real price of oil. See [22] for a survey of this literature. Recently, the Google Trends literature has become much broader: [23] quantified the degree to which Internet users worldwide seek more information about years in the future than years in the past, and found a strong correlation between the country's GDP and the predisposition of its inhabitants to look forward [24]. analyzed changes in Google query volumes for search terms related to finance and found patterns that may be interpreted as “early warning signs” of stock market moves [25]. proposed a novel approach to portfolio diversification using Google Trends, which is based on the idea that the popularity of a stock measured by search queries is correlated with the stock riskiness, while [26] analyzed the dynamic relationship between the BitCoin price and the interest in the currency measured by search queries on Google Trends and frequency of visits on the Wikipedia page on BitCoin.

In this perspective, we propose to use online search data for nowcasting and forecasting the monthly number of food stamps recipients: we justify this choice because the administrative burden for enrolling and remaining enrolled in the food stamps program is nontrivial, see e.g. [27], [28] and [3], and searching the web for information is one of the main strategies a potential applicant can do: for example, the most searched query related to the food stamps program for the US in the years 2004-2011 as provided by Google on 16/01/2012 was “*apply food stamps*”. Therefore, using Google online query statistics can provide real time information about the number of current and future food stamps recipients.

The first contribution of the paper is a detailed analysis of the main determinants of food stamps dynamics using the structural relationship identification methodology discussed by [29] and [30], which is a robust method of model selection in case of small samples. The second contribution of the paper is a large scale forecasting comparison with a set of almost 3000 models. In this regard, we computed nowcasts 1 step and 2 steps ahead, as well as out-of-sample forecasts up to 24 steps ahead, showing that models using Google data statistically outperform the competing models both for short term and long term forecasting. More specifically, we found that linear autoregressive models augmented with Google data definitively improve nowcasting food stamps data 2 months ahead, while simple linear models (eventually augmented with unemployment rates or initial claims data) are sufficient for nowcasting 1 month ahead. However, Google based linear models provided superior forecasts in case of 12 steps and 24 steps forecast ahead, whereas most nonlinear models performed very poorly, were computationally intensive, and in several cases did not reach numerical convergence. In this regard, the best models had specifications always close to the ARX(4) model (Auto-Regressive model with eXogenous variables). which was found using the structural relationship identification methodology in the in-sample analysis. Our results hold also with alternative Google keywords and with alternative out-of-sample periods which either include the NBER recession of the years 2007–2009 or start after the end of this recession. Moreover, they passed a falsification test recently proposed by [16]. Similar results were found when considering the directional accuracy of the models' forecasts and when forecasting at the state-level. We remark that the out-of-sample forecasting comparison was structured to replicate the situation that real forecasters face when they compute their forecasts, so that all exogenous variables (for example Google data) have to be predicted to forecast the endogenous variables of interest (in our case the number of food stamps) and avoid any look-ahead bias.

## Materials and Methods

### Data and In-Sample Analysis

The monthly number of individuals enrolled in the Food Stamps/SNAP program were collected from the USDA, for the period from October 1988 till May 2011, both at the US national level and at the state level. Unfortunately, these data included not only the standard income-based food stamps but also the so called *disaster food stamps*, which “… *provide replacement benefits for regular food stamp recipients who lose food in a disaster and extends benefits to many households which would not ordinarily be eligible but suddenly need food assistance*” (see the full details at http://frac.org/federal-foodnutrition-programs/snapfood-stamps/disaster-snapfood-stamps/). Following an interesting discussion with the people working at USDA who provided us with the data, we proceeded to clean the original data from the disaster food stamps for two main reasons:

- The two food stamps programs have very different economic rationale: the disaster food stamps are usually a very short term phenomenon which follows from natural disasters (floods, tornados, and so on), while food stamps for income reasons are a much more persistent process;
- Disaster food stamps create spikes/jumps in the data which can hinder considerably the estimation of any econometric models.

**Figure 2:**
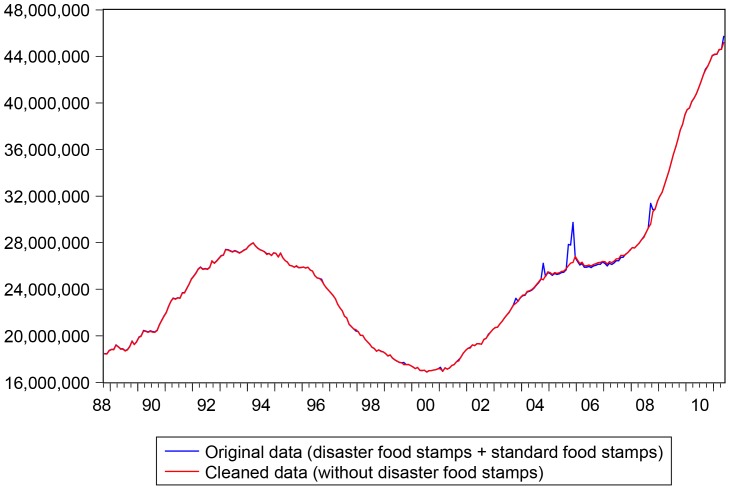
Original an cleaned food stamp data at the US national level. Sample: 1988M10 - 2011M5.

The cleaning process was very long, since the disaster food stamps were not in standardized format and were reported in different data type, so that the correction was made month by month, state by state, for all states, and with these data we then reconstructed the total number of food stamps recipients at the US national level for all months considered. The original and cleaned datasets are reported in Figure 2.

The spike in disaster food stamps following the havoc caused by hurricane Katrina is clearly visible.

**Figure 3:**
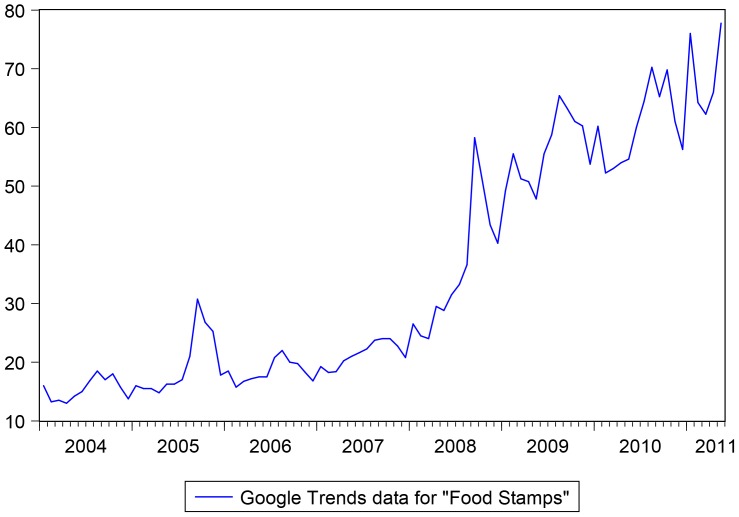
GI for the keywords “*food stamps*”. Sample: 2004M1 - 2011M5. Google data are registered trademarks of Google Inc., used with permission.

We then collected the GI for the keywords “*food stamps*” at the US national level for the period from January 2004 till May 2011. We remark that the GI is computed as the ratio of the search queries for a specific keyword (or group of keywords) relative to the total number of searches performed in the selected region at a given point of time, and then standardized between 0 and 100 (where the standardization is done over the whole time period). It is usually updated weekly, if not daily. The GI had a weekly frequency but was transformed into a monthly frequency to match food stamps data, see Figure 3.

Among the set of variables that we used to forecast food stamps, we also considered the unemployment rate. Its monthly data are available from January 1976 and can be downloaded from the U.S. Bureau of Labor Statistics, both at the US national and state level. This is one of the most frequently used variables used to model food stamps in the US, and was found able to explain a large part of the variation in food stamps dynamics, see e.g. [31], [3] and references therein. Moreover, in the fewer cases when forecasting was of concern, like [2], [10] and [11], the unemployment rate was one of the variables with the highest forecasting power [16]. found that models augmented with the GI for the keyword “jobs” significantly outperformed a very wide range of competing models when forecasting the US unemployment rate. Given this evidence and considering that the unemployment rate is one of the major predictor of the number of food stamps recipients, we also included this GI in our set of predictors. Finally, the monthly Initial Claims (IC) were also considered: they are available from January 1971 and can be downloaded from the US Department of Labor, both at the US national and state level. We employed this time series because it is a widely accepted leading indicator for the US unemployment rate, see [16] and references therein.

To analyze the food stamps dynamics, we employed the structural relationship identification methodology discussed by [29] and [30], which is specifically designed for small samples. The first step is to identify the order of integration using unit root tests: if all variables are stationary, VAR and VARX (Vector Autoregressive with exogenous variables) models are used. The second step determines the exogeneity of each variable using the sequential reduction method for weak exogeneity by [30], who consider weakly exogenous each variable for which the test is not rejected and re-test the remaining variables until all weakly exogenous variables are identified. For non-stationary variables, cointegration rank tests are employed to determine the presence of a long-run relationship among the endogenous variables: if this is the case, VECM or VECMX (Vector Error Correction model with exogenous variables) models are used, otherwise VAR or VARX models in differences are applied. The last step is to compute out-of-sample forecasts, see [29] for more details. However, our approach differs from the latter in that we employ unit root tests and cointegration tests allowing for structural breaks.

#### Stationarity

We analyzed the stationarity of food stamps data using a set of unit root tests allowing for structural break(s) because the food stamps legislation underwent a series of reforms during its history: the 1993 Mickey Leland Childhood Hunger Relief Act, the Personal Responsibility and Work Opportunity Reconciliation Act of 1996 (PRWORA), the 1996 Farm Bill, and the 2008 Farm Bill, just to name the most important, see [3] for an overview. Moreover, a global recession hit worldwide in 2007–2009, reaching the apex with the bankruptcy of Lehman Brothers. More specifically, we employed five unit root tests: the [32] unit root tests allowing for one and two breaks, respectively, and the Range Unit Root test (RUR) and the Forward-Backward RUR test suggested by [33], which are non-parametric tests robust against nonlinearities, error distributions, structural breaks and outliers. Furthermore, we also employ a periodic unit root test, given a mild presence of periodicity in the US food stamps data: if we perform a simple regression of the log returns of the monthly food stamps on 12 seasonal dummies over the full time sample, four seasonal dummies are statistically significant at the 1% level (using HAC robust standard errors) and the adjusted 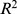 is 12%. This is a first-stage regression that was suggested by [34] to verify the potential presence of periodicity in the data: the mild value of the 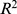 highlight the need to take periodicity into account. To test the null hypothesis of a periodic unit root, we follow the two-step strategy suggested by [35] and [34]: in the first step, a likelihood ratio test for testing a single unit root in a Periodic Auto-Regressive (PAR) model of order 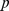 is performed (the order 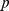 is chosen by using the Schwartz information criterion and checking that the residuals are approximately white noise); if the null of a periodic unit root cannot be rejected [35] and [34], suggest to test in a second step whether the process contain a non periodic unit root equal to 1 for all seasons. Since there is no version of this test with endogenous breaks, we estimated it both with the full sample starting in 1988, and with a smaller sample starting in 2008 to take the global financial crisis into account. As for the GIs, we want to remark that even though they are bounded between 0 and 100, this does not imply that they are stationary: for example, a random walk divided by its maximum value and multiplied by 100 (i.e. the procedure for computing the GI) remains non-stationary. Besides, the statistical effects of dividing the original search data for a specific keyword (which can be non stationary) by the total number of web searches in the same week and same area (which can be non stationary as well) are unknown, see also [21] for a discussion. The results of these tests for the log-transformed data of all variables are reported in Table 1 (the results for data in levels are similar and are not reported for sake of space - the software used to compute these tests is discussed in Software Description S1).

**Table 1:** Unit root tests.

	**RUR**	**FB RUR**	**LS (1 break)**	**LS (2 breaks)**
	*(* 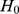 *: unit root)*	*(* 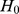 *: unit root)*	*(* 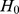 *: unit root)*	*(* 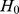 *: unit root)*
*Unemployment*	1.34	1.85	−3.26 [1997M4]	−4.06[1996M4,2008M8]
*Initial Claims*	0.85*	1.07*	−3.94[2007M9]	−5.22[1992M11,200810]
*GI Food Stamps*	1.60	2.94*	−4.53*[2008M7]	−5.60*[2006M8,2008M8]
*GI Jobs*	1.17*	1.58	−5.52*[2007M4]	−6.48*[2007M3,2009M9]
*Food Stamps*	5.16*	7.86*	−3.10[1998M10]	−3.72[1992M5,1999M3]
	**Periodic u.r. test - Sample: 1988–2011**		**Periodic u.r. test – Sample: 2008–2011**	
	**1nd step (LR stat.)**	**2nd step (p-value)**	**1nd step (LR stat.)**	**2nd step (p-value)**
	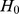 *: periodic u.r.*	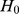 *: non periodic u.r.*	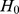 *: periodic u.r.*	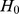 *: non periodic u.r.*
*Unemployment*	0.02	**0.00**	31.55*	/
*Initial Claims*	15.05*	/	3.94*	/
*GI Food Stamps*	NA	NA	1.41	**0.01**
*GI Jobs*	NA	NA	6.46*	/
*Food Stamps*	20.20*	/	8.06*	/

Unit root tests: RUR  =  Range Unit Root test by [33]; FB  =  Forward-Backward RUR test by [33]; LS = unit root test with breaks by [32] - the estimated break dates are reported in brackets. The second step for the periodic unit root tests by [35] and [34] is performed only if the first step did not reject the null hypothesis of a periodic unit root. P-values smaller than 0.05 are in bold font. * Significant at the 5%, level.

The evidence emerging from the (non-periodic) unit root tests is somewhat mixed but points to stationarity for almost all time series, with structural breaks at the end of the ’90s and at the beginning of the global financial crisis in 2007–2008. This evidence is also indirectly confirmed by the periodic unit root tests, whose outcomes changes substantially if the sample used changes, particularly for the unemployment rate. The latter data is probably the one which has the more mixed evidence: in this regard, we are aware of the very hot discussion about the stationarity of unemployment rates and we refer to [36] and [16] for a review of this debate. Given this evidence, we decided to follow a neutral approach and in the forecasting section we compared both models with the data in levels and models with first-differenced data.

#### Weak exogeneity and Cointegration

The next step in the structural relationship identification methodology suggested by [29] is to determine the exogeneity of each variable using the sequential reduction method for weak exogeneity proposed by [30], which is specifically designed for small samples: once a weakly exogenous variable is found, the remaining variables are re-tested until all weakly exogenous variables are identified. Given the previous mixed evidence of stationarity, we employed both the standard Wald test using a VAR model in levels with centered seasonal dummies, and the Wald test proposed by [37] which is valid in case the variables may be integrated or cointegrated of an arbitrary order (we included centered seasonal dummies because they sum to zero over time and therefore do not affect the asymptotic distributions of the tests, see [38] and [39] for details). This last approach requires, first, to determine the appropriate maximum lag length 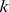 for the variables in the VAR in levels using information criteria; then, to estimate a 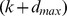 th-order VAR where 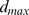 is the maximum order of integration that we suspect for our group of time-series. Finally, [37] show that we can test linear or nonlinear restrictions on the first 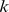 coefficient matrices using standard asymptotic theory, while the coefficient matrices of the last 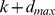 lagged vectors have to be ignored. We chose 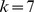 after looking at a battery of information criteria (AIC, BIC, Hannan-Quinn, Forecast Prediction Error) and checking that the residuals behave approximately as a multivariate white noise. Moreover, in our case 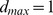. The results of the sequential reduction method for weak exogeneity using the standard Wald test with a VAR(7) and the Wald test proposed by [37] with a VAR(8) are reported in Table 2. Variables whose Wald test has a p-value larger than 5% are considered weakly exogenous and are excluded from further testing.

**Table 2:** P-values of sequential tests for weak exogeneity.

	Wald test		Toda-Yamamoto	
	1st step	2nd step	1st step	2nd step
*Unemployment*	**0.01**	**0.00**	0.05	/
*Initial Claims*	**0.00**	**0.00**	**0.00**	0.07
*GI Food Stamps*	0.58	/	0.79	/
*GI Jobs*	**0.01**	**0.00**	0.26	/
*Food Stamps*	**0.00**	**0.00**	**0.00**	**0.00**

P-values of sequential tests for weak exogeneity: standard Wald test and Wald test using the approach by [37]. P-values smaller than 0.05 are in bold font.

The results of the two approaches differ considerably: for the standard Wald test, only the GI for the keyword “food stamps” is weakly exogenous, while for the Toda and Yamamoto approach all four predictors are weakly exogenous (the unemployment rate and the two GIs in the first step, while the initial claims in the second step). It may well be the case that the global financial crisis in 2008, which was a significant break in the previous unit root tests, could be one of the main reasons of these different results. Unfortunately, our sample is too short to estimate VAR(7) and VAR(8) models starting in 2008.

Given the somewhat mixed evidence about stationarity, we proceeded nonetheless to test for cointegration among our five variables as a potential cross-check: if the variables are all stationary, the multivariate cointegration tests should find a number of cointegration relationships equal to the number of the variables examined. In this regard, the Johansen cointegration tests can be used as panel unit root tests, as discussed by [40] and [41]. More specifically, we used a set of cointegration tests allowing for the presence of structural break(s):

- [42] single-equation cointegration test allowing for one endogenous break;
- [43] single-equation cointegration test allowing for two endogenous breaks;
- [44] multivariate test allowing for the presence of one or two exogenous break(s), where the dates of the breaks are the ones selected by the [42] and [43] tests, respectively.

For sake of generality, we also considered the single-equation test by [45] and multivariate cointegration test by [38], both of them without breaks. The main advantage of single-equation approaches is that they allow for endogenous breaks. However, these tests are not suitable when the right hand variables in the cointegration vector are not weakly exogenous (which is not our case, according to the approach by [37]) and when there are more than one cointegrating vector. The only problem with the multivariate tests by [44] is that they allow only for exogenous breaks. Therefore, we followed a 2-step strategy: we first estimated the single-equation approaches by [42] and [43] to have an indication of the structural breaks dates, and we then used these dates to compute the multivariate tests by [44], see Table 3.

**Table 3:** Single-equation and multivariate cointegration tests with and without structural break(s).

Single-equation cointegration tests				
Engle and Granger (1987)	Gregory and Hansen (1996)		Hatemi (2008)	
No breaks	1 (endogenous) break		up to 2 (endogenous) breaks	
*Tau statistic*	*Z-t statistic*	*Break date*	*Z-t statistic*	*Break dates*
−3.83	−4.82	2009M11	−5.29	2006M1 2009M1

Single-equation and multivariate cointegration tests with and without structural break(s). The null hypothesis for all tests is the absence of cointegration. All the tests considered the case of no deterministic trend in the data and an intercept in the cointegration equation (CE), centered seasonal dummies outside the CE, while the number of lags is chosen using the Schwartz criterion. The tests allowing for break(s) considered the case of a level shift. * Significant at the 5% level.

All single-equation tests do not reject the null of no cointegration, while the Johansen tests allowing for break(s) found evidence of five CEs in a system of five variables, which means that all the five variables are stationary. Only the Johansen test with no breaks found evidence of a cointegrated system with 4 CEs, but the presence of a break during the global financial crisis suggests some caution when interpreting this last result. Therefore, this evidence of absence of cointegration and stationary variables is consistent with the previous weak exogeneity tests and unit root tests. We remark that periodic cointegration tests using all variables could not be implemented due to the high number of parameters to be estimated. This “curse of dimensionality” is a well known problem for this kind of tests, see [34] for more details.

Finally, the values of the significant parameters at the 5% level for the equation of the monthly number of food stamps recipients in log-levels are reported in Table 4. A battery of misspecification and stability tests is also reported in the same table.

**Table 4:** Estimated coefficients in the equation of food stamps recipients (left block) and misspecification and stability tests (right block).

*Regressors*	*Coeff.*	*T-stat*	*Tests*	*p-value*
log(Food stamps(-1))	0.59	5.40	Ljung-Box(12)	0.52
log(Food stamps(-2))	0.30	2.31	Ljung-Box(24)	0.65
log(Food stamps(-3))	0.29	2.22	Ljung-Box(12) res. sq.	0.79
log(Food stamps(-4))	-0.23	-2.25	Ljung-Box(24) res. sq.	0.79
log(Unemployment rate)	0.02	3.13	ARCH(12)	0.89
log(GI - Food Stamps)	0.01	3.96	ARCH(24)	0.98
log(GI - Jobs)	0.02	2.03	Jarque-Bera	**0.00**
constant	0.87	4.63	RESET	0.56
S1	−0.02	−5.74	BDS (dim = 2)	0.12
S2	−0.02	−8.07	BDS (dim = 6)	**0.00**
S3	−0.01	−4.37	OLS-CUSUM	0.99
S4	−0.02	−4.44	Rec-CUSUM	0.06
S5	−0.01	−3.43	OLS-MOSUM	0.51
S6	−0.02	−4.04	Rec-MOSUM	0.39
S7	−0.01	−3.95	Andrews max-F	**0.03**
S8	−0.01	−3.89	Andrews exp-F	0.22
S9	−0.01	−4.64	Andrews ave-F	0.09
S10	−0.01	−3.69	Optimal n. breakpoints (BIC)	0
S11	−0.01	−4.36	Optimal n. breakpoints (LWZ)	0

Estimated coefficients in the equation of food stamps recipients (left block) and misspecification and stability tests (right block). Sample: 2004M1- 2011M05. P-values smaller than 0.05 are in bold font.

*Misspecification tests*: the [46] statistics for testing the absence of autocorrelation up to order 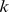 in the models' residuals and residuals squared; the Lagrange multiplier test for Auto-Regressive Conditional Heteroskedasticity (ARCH) in the residuals proposed by [47]; the [48] test for checking whether a time series is normally distributed; the REgression Specification Error Test (RESET) proposed by [49], which is a general test for incorrect functional form, omitted variables, and correlation between the regressors and the error term; the BDS test by [50] to test whether the residuals are independent and identically distributed (iid) and which is robust against a variety of possible deviations from independence, including linear dependence, non-linear dependence, or chaos.

*Stability tests*: the test for parameter instability by [51] which is based on the CUmulative SUM of the recursive residuals (Rec-CUSUM); [52] suggested to modify the previous structural change test and use the cumulative sums of the common OLS residuals (OLS-CUSUM). [53] proposed a structural change test which analyzes moving sums of residuals (MOSUM) instead of cumulative sums. We remark that a unifying view of the previous structural change tests within a generalized M-fluctuation test framework was proposed by [54] and [55]. [56] was the first to suggest an F-test for structural change when the break point is known: [57] and [58] extended the Chow test by computing the F statistics for all potential break points and suggested three different test statistics, the sup-F, the ave-F and the exp-F, which are based on Wald, Lagrange Multiplier or Likelihood Ratio statistics respectively, in a very general class of models fitted by Generalized Method of Moments. See [59] for a review and a step-by-step description of stability tests using R software. Besides, [60], following [61], suggested to find the optimal number of breakpoints by optimizing the Bayesian Information Criterion (BIC) and the modified BIC by [62] (LWZ, 1997).

The GIs for the keywords “food stamps” and “jobs” and the unemployment rate have all a positive effect on the number of food stamps recipients: an increase in these variables, increase the the number of food stamps. Instead, the number of initial claims was found not significant at the 5% level and therefore was not reported in Table 4. The sum of the autoregressive coefficients is 0.94, somewhat close to 1, thus confirming the mixed evidence about stationarity which emerged from unit root tests in Table 1. As previously highlighted by the stationarity and cointegration tests, this may be due to a break in 2008–2009. However, the parameter stability tests do not signal strong evidence of model instability, and similarly the misspecification tests do not show any serious problem in the model's residuals, except for some nonlinearity and the lack of residuals' normality. The latter issue suggests caution when reading the previous t-statistics, considering that our sample consists of 89 observations: one possibility could be to resort to bootstrap methods or to use robust estimation methods. Given that the focus of this work is forecasting, we preferred to deal with this issue by comparing the forecasting performances of a very large set of model specifications: with different number of autoregressive lags, with and without seasonal dummies, with and without Google indexes, with and without weakly exogenous regressors, with data in levels and in first differences. Such an approach allows us to take a neutral stance towards the competing models and avoid any form of look-ahead bias. Moreover, we could analyze the models' behavior during the potential structural break caused by the global financial crisis.

### Forecasting Models

Food stamps data are reported with a 2-month lag: the latest data relative to month 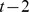 are issued in the first working days of month 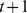. For example, in the first days of January 2011, the data about October 2010 were released. As discussed before, the data are released with a 2-month delay due to the high noise in the initial data. Therefore, in order to “*nowcast*” the value of food stamps for November 2010 (i.e. month 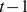) and December 2010 (i.e. month 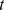), we can use the Google data up to December 2010 (i.e. month 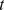), the initial claims up to December 2010 (i.e. month 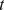), and the unemployment rate up to November 2010 (i.e. month 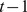), since it is released with 1-month lag. Besides nowcasting, we also consider forecasting monthly food stamps 12 months ahead and 24 months ahead, given its importance for policy makers and public planners when preparing public budgets.

The regressors used to explain the dynamics of the monthly food stamps are the aforementioned monthly Google indexes for the keyword “food stamps” and “jobs” [in the following tables, GI(J.&F.S.) will represent the case where both the GIs for “food stamps” and “jobs” are present as regressors, GI(F.S.) the case with only the GI for “food stamps”, whereas GI(J.) the case with only the GI for “jobs”], the monthly unemployment rates (UR) and the monthly initial claims (IC): these regressors may enter the equation simultaneously as weakly exogenous variables (in case of GIs and IC), with 1 lag (in case of the UR, since it is released with 1-month lag), simultaneously and with lags up to order 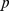 (in case of GIs and IC) and with lags up to order 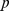 (in case of the UR).

Models without Google data were estimated on two different time samples (1988M10-2011M5, 2004M1-2011M5) to consider the effects of potential structural breaks. In this regard [63], showed that in a regression with a single break, the optimal window for estimation includes all of the observations after the break, plus a limited number of observations before the break, and similar results also hold for multiple breaks (in this case the last break has to be considered): since the evidence so far points to a break at the end of the ’90s and at the beginning of the global financial crisis, using a second estimation sample starting from 2004 should be a good compromise between efficiency and bias. Moreover, we also considered four possible data transformation: the original data in levels, the log-transformed data, the first differences and the first differences in logs (i.e. the log-returns). This was done to consider both stationarity and non stationarity, as well as simple nonlinearity captured by the log transformation.

The wide range of models we considered for nowcasting and forecasting can be grouped into five general classes:

- ***Linear models***. In this class, we included three types of models:
  - AR(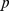) models, eventually augmented with additional regressors, simultaneous and/or lagged as discussed above (i.e. ARX(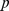) models):

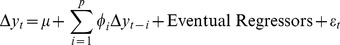

  - ARMA(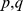) models, eventually augmented with additional regressors, simultaneous and/or lagged (i.e. ARMAX(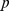) models):

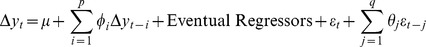

  - AR(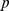) models with seasonal dummies, eventually augmented with additional regressors, simultaneous and/or lagged (i.e. AR-SD-X(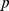) models):

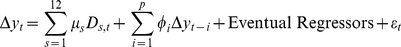

- ***Periodic models***. Four types of models were considered:
  - PAR(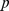) models, eventually augmented with additional regressors, simultaneous and/or lagged (i.e. PAR-X(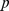) models):

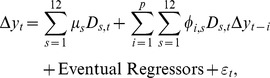

  - PAR(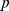) models with periodic trends, eventually augmented with additional regressors, simultaneous and/or lagged (i.e. PAR-T-X(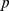) models):

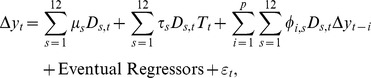
where 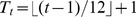 with 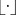 the integer function, represents an annual linear deterministic trend.

  - PAR(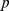)-ARCH(1) models, eventually augmented with additional regressors, simultaneous and/or lagged (i.e. PAR-X(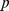)-ARCH(1) models):

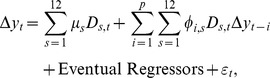


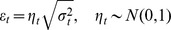


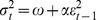

  - Periodic Error Correction (PEC) models: we considered the case of periodic cointegration when the variables have a non-periodic unit root:

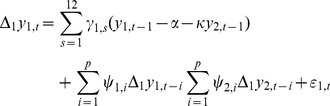
(1)where 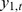 is the number of food stamps recipients while 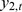 is a set of regressors, see [34] for more details about this single-equation cointegration model. For computational tractability, only the two cases of cointegration between food stamps and GIs, and cointegration between food stamps, UR and IC were considered. Considering the relative small out-of-sample (more below) and the number of variables involved, we considered PEC(1,12) models.

- ***Multivariate models***. Three types of models were considered in this class:
  - Vector Auto-Regressive (VAR) models: given the sample dimension and the number of variables, we considered only trivariate VAR models including either food stamps and the two GIs, or food stamps and the UR and the IC.
  - Vector Error Correction (VEC) models, where all potential cointegration relationship between food stamps and the four regressors (UR, IC, GIs for “food stamps” and “jobs”) were explored. We considered the case of no trend in data and no trend in cointegration relation, but with intercepts in the cointegration relations:

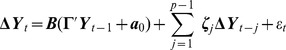
where 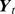 is an 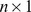 vector process, 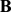 is an 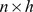 matrix of loadings (or correction factors), 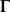 is an 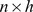 matrix containing the cointegrating vectors, and 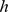 is the number of cointegrating relationships, i.e. the cointegration rank, see [38] for details. Similarly to PEC models, we considered VEC(1,12) models, with only 1 CE for computational tractability.

-
  - Bayesian Vector Auto-Regressive (BVAR) models: when there are a lot of variables and lags, estimating VAR models can be challenging, if not impossible. One way to solve this issue is to shrinkage the parameters by using Bayesian methods. Bayesian VAR models has recently enjoyed a large success in macroeconomic forecasting, see [64] for a recent review and [21] for a recent application with Google data. More specifically, we used the so called Litterman/Minnesota prior, which is a common choice in empirical applications due to its computational speed and forecasting success, see [65], [66] and [64] for more details.
- ***Nonlinear models***. Four types of models were considered (see [21] for a recent application of these nonlinear models to forecast the real price of oil using Google data - the nonlinear model proposed by [8] to forecast food stamps caseloads was not considered because we did not have neither the monthly data relative to the new entries and exits for each state for the income-based food stamps program, nor the monthly data relative to the new entries and exits for the disaster food stamps):
  - Self-Exciting Threshold AutoRegressive (SETAR) models with 2 regimes:

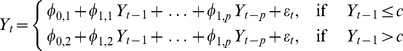
where 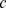 is a threshold to be estimated and which identifies the two regimes.

  - Logistic smooth transition autoregressive (LSTAR) models, which are a generalization of the SETAR model:

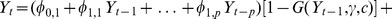


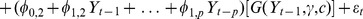
where 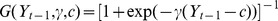 is the first order logistic transition function, bounded between 0 and 1, 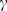 is the slope parameter and 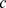 is the location parameter. In contrast with the SETAR models, the LSTAR model assumes that the change between the two regimes is gradual and smooth. This model belongs to the class of Smooth Transition AR models, see [67] for details.

  - Neural Network (NNET) models with linear output, defined as follows:

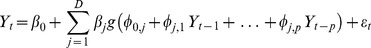
where 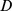 is the number of hidden units and 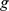 is the activation function, given by the logistic function. See [68] (chapter 8) and [69] (chapter 5) for details. In this case, we chose the number of hidden units 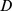 to be 3 based on information criteria.

- Additive Autoregressive models (AAR), also known as generalized additive models, since they combines generalized linear models and additive models:



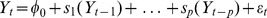
where 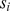 are smooth functions represented by penalized cubic regression splines, see [70] for details.

- Last, but not least, ***the Random Walk with Drift model***, which is the classical benchmark model for economic and financial forecasting: 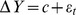.

The full list of the 2890 models used in our forecasting exercise is reported in the Tables 5–6. Finally, we remark that our forecasting comparison was structured to replicate the situation that real forecasters face when they compute their forecasts, and they have to use only the information available at each point in time: therefore, predictions of all the exogenous variables (for example Google data) have to be computed to forecast the endogenous variables of interest (in our case, the number of food stamps recipients). To satisfy this criterion and avoid any problem of look-ahead bias, we had to choose a forecasting model for the unemployment rate, the initial claims and the Google indexes: based on information criteria and residuals properties, we selected a PAR(1) model for the monthly unemployment rate and initial claims in logs, and an AR(12) model for the log-returns of Google indexes. The forecasts of these exogenous variables then served as inputs in the forecasting models for monthly food stamps data. Clearly, we could have considered a range of models for the exogenous regressors, but this would have increased exponentially the total number of models, making the forecasting exercise computationally untractable. Therefore, we leave this issue as an avenue for further research.

**Table 5:** Models used for nowcasting and forecasting: Linear and Periodic models.

	LAGS	Additional regressors														Time Samples		Data Transformations				Total models
		NO REG.	*GI(FS)*		*GI(JOBS)*		*GIs(JOBS & FS)*		*UR*		*IC*		*GI(FS), UR, IC*	*G(JOBS), UR, IC*	*GI(JOBS & FS), UR, IC*	1988M10-2011M5	2004M1-2011M5	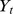	log( 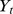 )	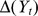	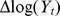	(sum by row)
Regressors:			*sim.*	*sim. and*	*sim.*	*sim. and*	*sim.*	*sim. and*	*lag1*	*lagged*	*sim.*	*sim. and*	*sim. +*	*sim. +*	*sim. +*							
sim./lagged				*lagged*		*Lagged*		*lagged*				*lagged*	*lag1*	*lag1*	*lag1*							
AR(p)	up to 12	yes														yes	yes	yes	yes	yes	yes	12 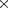 4 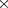 2 = 96
	up to 12		yes														yes	yes	yes	yes	yes	12 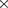 4 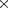 1 = 48
	up to 12			yes													yes	yes	yes	yes	yes	12 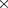 4 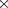 1 = 48
	up to 12				yes												yes	yes	yes	yes	yes	12 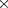 4 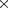 1 = 48
	up to 12					yes											yes	yes	yes	yes	yes	12 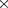 4 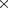 1 = 48
	up to 12						yes										yes	yes	yes	yes	yes	12 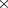 4 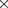 1 = 48
	up to 12							yes									yes	yes	yes	yes	yes	12 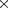 4 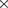 1 = 48
	up to 12								yes							yes	yes	yes	yes	yes	yes	12 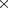 4 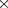 2 = 96
	up to 12									yes						yes	yes	yes	yes	yes	yes	12 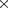 4 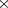 2 = 96
	up to 12										yes					yes	yes	yes	yes	yes	yes	12 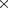 4 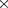 2 = 96
	up to 12											yes				yes	yes	yes	yes	yes	yes	12 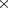 4 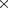 2 = 96
	up to 12												yes				yes	yes	yes	yes	yes	12 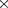 4 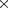 1 = 48
	up to 12													yes			yes	yes	yes	yes	yes	12 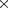 4 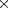 1 = 48
	up to 12														yes		yes	yes	yes	yes	yes	12 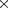 4 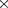 1 = 48
ARMA(p, q)	up to 12	yes														yes	yes	yes	yes	yes	yes	12 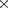 4 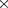 2 = 96
	up to 12		yes														yes	yes	yes	yes	yes	12 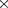 4 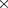 1 = 48
	up to 12				yes												yes	yes	yes	yes	yes	12 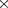 4 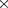 1 = 48
	up to 12						yes										yes	yes	yes	yes	yes	12 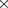 4 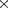 1 = 48
	up to 12								yes							yes	yes	yes	yes	yes	yes	12 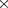 4 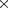 2 = 96
	up to 12										yes					yes	yes	yes	yes	yes	yes	12 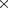 4 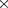 2 = 96
	up to 12												yes				yes	yes	yes	yes	yes	12 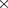 4 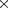 1 = 48
	up to 12													yes			yes	yes	yes	yes	yes	12 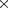 4 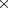 1 = 48
	up to 12														yes		yes	yes	yes	yes	yes	12 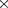 4 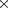 1 = 48
AR(p)	up to 12	yes														yes	yes	yes	yes	yes	yes	12 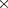 4 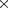 2 = 96
+ seasonal	up to 12		yes														yes	yes	yes	yes	yes	12 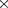 4 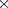 1 = 48
constants	up to 12				yes												yes	yes	yes	yes	yes	12 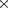 4 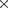 1 = 48
	up to 12						yes										yes	yes	yes	yes	yes	12 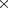 4 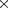 1 = 48
	up to 12								yes							yes	yes	yes	yes	yes	yes	12 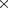 4 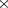 2 = 96
	up to 12										yes					yes	yes	yes	yes	yes	yes	12 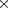 4 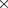 2 = 96
	up to 12												yes				yes	yes	yes	yes	yes	12 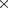 4 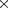 1 = 48
	up to 12													yes			yes	yes	yes	yes	yes	12 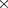 4 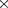 1 = 48
	up to 12														yes		yes	yes	yes	yes	yes	12 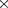 4 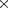 1 = 48
PAR(p)	up to 3	yes														yes	yes	yes	yes	yes	yes	3 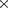 4 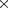 2 = 24
	up to 3		yes														yes	yes	yes	yes	yes	3 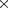 4 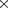 1 = 12
	up to 3			yes													yes	yes	yes	yes	yes	3 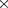 4 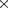 1 = 12
	up to 3				yes												yes	yes	yes	yes	yes	3 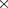 4 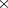 1 = 12
	up to 3					yes											yes	yes	yes	yes	yes	3 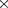 4 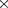 1 = 12
	up to 3						yes										yes	yes	yes	yes	yes	3 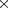 4 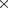 1 = 12
	up to 3							yes									yes	yes	yes	yes	yes	3 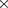 4 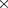 1 = 12
	up to 1								yes							yes	yes	yes	yes	yes	yes	1 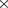 4 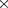 2 = 8
	up to 3									yes						yes	yes	yes	yes	yes	yes	3 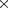 4 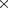 2 = 24
	up to 1										yes					yes	yes	yes	yes	yes	yes	1 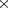 4 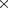 2 = 8
	up to 3											yes				yes	yes	yes	yes	yes	yes	3 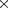 4 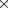 2 = 24
	up to 1												yes				yes	yes	yes	yes	yes	1 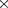 4 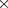 1 = 4
	up to 1													yes			yes	yes	yes	yes	yes	1 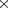 4 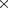 1 = 4
	up to 1														yes		yes	yes	yes	yes	yes	1 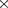 4 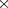 1 = 4
PAR(p)	up to 3	yes														yes	yes	yes	yes	yes	yes	3 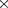 4 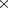 2 = 24
+ periodic	up to 3		yes														yes	yes	yes	yes	yes	3 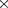 4 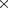 1 = 12
trends	up to 3				yes												yes	yes	yes	yes	yes	3 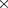 4 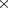 1 = 12
	up to 3						yes										yes	yes	yes	yes	yes	3 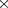 4 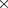 1 = 12
	up to 1								yes							yes	yes	yes	yes	yes	yes	1 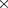 4 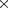 2 = 8
	up to 1										yes					yes	yes	yes	yes	yes	yes	1 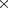 4 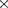 2 = 8
	up to 1												yes				yes	yes	yes	yes	yes	1 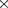 4 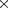 1 = 4
	up to 1													yes			yes	yes	yes	yes	yes	1 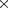 4 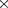 1 = 4
	up to 1														yes		yes	yes	yes	yes	yes	1 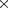 4 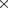 1 = 4
PAR(p)	up to 3	yes														yes	yes	yes	yes	yes	yes	3 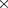 4 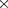 2 = 24
+ ARCH(1)	up to 3			yes													yes	yes	yes	yes	yes	3 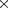 4 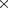 1 = 12
	up to 3									yes						yes	yes	yes	yes	yes	yes	3 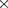 4 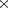 2 = 24
	up to 3											yes				yes	yes	yes	yes	yes	yes	3 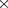 4 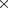 2 = 24
PEC	1,12		yes														yes	yes	yes			1 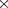 2 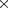 1 = 2
PEC	1,12								yes							yes	yes	yes	yes			1 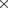 2 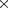 2 = 4
PEC	1,12										yes					yes	yes	yes	yes			1 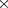 2 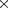 2 = 4
PEC	1,12								yes		yes					yes	yes	yes	yes			1 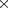 2 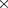 2 = 4
PEC	1,12						yes										yes	yes	yes			1 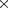 2 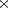 1 = 2

**Table 6:** Models used for nowcasting and forecasting: Multivariate models, Nonlinear models and Random Walk with drift.

		Additional regressors																	Time Samples		Data Transformations				Row Total
MODELS	LAGS	NO REG.	*GI (JOBS & FS)*	*UR*	*IC*	*UR&IC*	*GI (FS)*	*GI (FS), UR*	*GI (FS), IC*	*GI (FS), UR, IC*	*GI (JOBS)*	*GI (JOBS), UR*	*GI (JOBS), IC*	*GI (JOBS), UR, IC*	*GI (JOBS & FS)*	*GI (JOBS & FS), UR*	*GI (JOBS & FS), IC*	*GI (JOBS & FS), UR, IC*	1988M10-2011M5	2004M1-2011M5	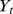	log 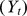	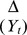	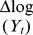	models
VAR	1–7		yes																	yes	yes	yes			1 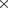 2 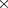 1 = 2
VAR	1–7					yes													yes	yes	yes	yes			2 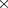 2 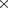 1 = 4
VAR	1–6		yes																	yes			yes	yes	1 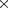 2 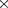 1 = 2
VAR	1–6					yes													yes	yes			yes	yes	2 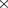 2 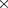 1 = 4
BVAR	1–7						yes													yes	yes	yes			1 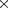 2 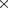 1 = 2
BVAR	1–6						yes													yes			yes	yes	1 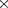 2 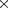 1 = 2
BVAR	1,12						yes													yes	yes	yes	yes	yes	1 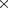 4 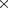 1 = 4
BVAR	1–7			yes	yes														yes	yes	yes	yes			2 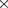 2 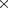 1 = 4
BVAR	1–6			yes	yes														yes	yes			yes	yes	2 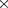 2 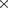 1 = 4
BVAR	1,12			yes	yes														yes	yes	yes	yes	yes	yes	2 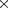 4 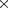 1 = 8
BVAR	1–7		yes																	yes	yes	yes			1 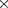 2 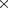 1 = 2
BVAR	1–6		yes																	yes			yes	yes	1 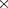 2 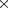 1 = 2
BVAR	1,12		yes																	yes	yes	yes	yes	yes	1 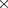 4 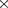 1 = 4
VEC	1,12			yes															yes	yes	yes	yes			2 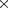 2 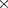 1 = 4
VEC	1,12				yes														yes	yes	yes	yes			2 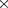 2 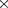 1 = 4
VEC	1,12					yes													yes	yes	yes	yes			2 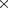 2 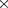 1 = 4
VEC	1,12						yes													yes	yes	yes			1 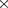 2 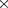 1 = 2
VEC	1,12							yes												yes	yes	yes			1 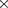 2 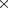 1 = 2
VEC	1,12								yes											yes	yes	yes			1 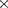 2 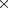 1 = 2
VEC	1,12									yes										yes	yes	yes			1 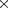 2 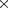 1 = 2
VEC	1,12										yes									yes	yes	yes			1 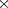 2 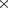 1 = 2
VEC	1,12											yes								yes	yes	yes			1 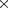 2 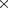 1 = 2
VEC	1,12												yes							yes	yes	yes			1 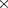 2 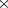 1 = 2
VEC	1,12													yes						yes	yes	yes			1 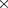 2 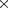 1 = 2
VEC	1,12														yes					yes	yes	yes			1 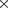 2 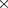 1 = 2
VEC	1,12															yes				yes	yes	yes			1 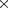 2 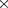 1 = 2
VEC	1,12																yes			yes	yes	yes			1 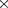 2 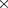 1 = 2
VEC	1,12																	yes		yes	yes	yes			1 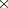 2 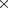 1 = 2
SETAR	up to 12	yes																	yes	yes	yes	yes	yes	yes	12 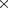 4 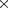 2 = 96
LSTAR	up to 12	yes																	yes	yes	yes	yes	yes	yes	12 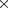 4 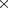 2 = 96
AAR	up to 12	yes																	yes	yes	yes	yes	yes	yes	12 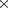 4 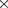 2 = 96
NNET	up to 12	yes																	yes	yes	yes	yes	yes	yes	12 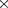 4 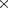 2 = 96
RW		yes																	yes	yes				yes	1 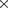 2 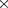 1 = 2
**GRAND TOTAL: 2890 MODELS**																									

## Results

### Out-of-Sample Forecasting Analysis

We used the data between 1988M10 and 2007M2 as the first initialization sample for the models without GIs, while we used the initialization sample 2004M1-2007M2 for the models with GIs and for those models without GIs but estimated on a shorter sample. The evaluation period ranged from 2007M3 till 2011M5 and was used to compare the nowcasts 1 step and 2 steps ahead, as well as the forecasts 12 steps and 24 steps ahead. The total number of models using Google data among the Top 100 models in terms of Root Mean Square Error (RMSE) is reported in Table 7, while Table 8 reports the ranking of the best models within each class according to the RMSE. Finally, the top 10 models in terms of the RMSE for nowcasting and forecasting are reported in Tables 9–10.

**Table 7:** Number of models with Google data out of the Top 100 models according to the RMSE.

	Nowcasting 1 s.a.	Nowcasting 2 s.a.	Forecasting 12 s.a.	Forecasting 24 s.a.
RMSE	41	90	92	91

**Table 8:** Ranking of the best models within each class according to the RMSE.

	Type of	Nowcasting	Nowcasting	Forecasting	Forecasting
	models	1 s.a.	2 s.a.	12 s.a.	24 s.a.
*Linear*	AR w/GI	17	81	127	177
*models*	AR w/o GI	5	75	75	128
	ARMA w/GI	138	51	74	153
	ARMA w/o GI	1	113	87	123
	AR + s.d. w/GI	17	1	1	1
	AR + s.d. w/o GI	14	38	180	180
*Periodic*	PAR w/GI	2530	2364	17	41
*models*	PAR w/o GI	444	948	690	822
	PAR+p.t. w/GI	2632	2623	959	145
	PAR+p.t. w/o GI	391	613	377	463
	PAR-ARCH w/GI	2635	2514	555	159
	PAR-ARCH w/o GI	1138	1459	610	836
	PEC w/GI	2538	2547	53	44
	PEC w/o GI	1783	2442	72	703
*Multivariate*	VAR w/GI	236	441	2053	2462
*models*	VAR w/o GI	293	345	61	229
	VEC w/GI	102	194	856	1518
	VEC w/o GI	209	367	257	627
	BVAR w/GI	7	370	515	411
	BVAR w/o GI	197	907	925	1301
*Nonlinear*	SETAR	Not converged	Not converged	Not converged	Not converged
*models*	LSTAR	716	1144	410	137
	NNET	1359	1595	923	797
	AAR	383	704	82	40
*Random W.*	RW	2562	2585	1847	1183

**Table 9:** Top 10 models in terms of RMSE - baseline case. Nowcasting: 1 step and 2 steps ahead.

1 STEP ahead (baseline case)		2 STEPS ahead (baseline case)	
Top 10 models	RMSE	Top 10 models	RMSE
ARMA(10,10) dlog 1988	159024	AR(3)+S.D.+UR+IC+GI(J.&F.S.) log 2004	211508
ARMA(10,10) + UR dlog 1988	160819	AR(8)+S.D.+GI(F.S.) lev 2004	211784
ARMA(12,12) dlog 1988	161311	AR(7)+ S.D.+GI(F.S.) lev 2004	211843
ARMA(11,11) + UR diff 1988	162494	AR(2)+S.D.+UR+IC+GI(J.&F.S.) log 2004	212644
AR(12) + IC(sim+lags) dlog 1988	164194	AR(7)+S.D.+GI(J.&F.S.) lev 2004	212878
ARMA(12,12) + IC diff 1988	165172	AR(4)+ S.D.+GI(F.S.) lev 2004	214086
BVAR(1,12) FS+GI(F.S.) lev 2004	165369	AR(4)+S.D.+GI(J.&F.S.) lev 2004	215379
BVAR(1,12) FS+UR+IC+GI(F.S.) lev 2004	165531	AR(8)+S.D.+GI(J.&F.S.) lev 2004	215468
ARMA(12,12) + UR dlog 1988	166215	AR(5)+ S.D.+GI(F.S.) lev 2004	216076
ARMA(11,11) dlog 1988	167503	AR(6)+S.D.+UR+IC+GI(J.&F.S.) log 2004	216667

In each row, the following information is reported: the model, the number of lags, (eventual) exogenous regressors, the data transformation, the first year of the estimation sample.

**Table 10:** Top 10 models in terms of RMSE - baseline case. Forecasting: 12 steps and 24 steps ahead.

12 STEPS ahead (baseline case)		24 STEPS ahead (baseline case)	
Top 10 models	RMSE	Top 10 models	RMSE
AR(2)+S.D.+UR+IC+GI(J.) log 2004	1495400	AR(6)+S.D.+UR+IC+GI(J.&F.S.) log 2004	3775883
AR(5)+S.D.+UR+IC+GI(J.&F.S.) log 2004	1527588	AR(5)+S.D.+UR+IC+GI(J.&F.S.) log 2004	3777359
AR(3)+S.D.+UR+IC+GI(J.) log 2004	1534364	AR(2)+S.D.+UR+IC+GI(J.&F.S.) log 2004	3830094
AR(4)+S.D.+UR+IC+GI(J.&F.S.) log 2004	1544779	AR(2)+S.D.+UR+IC+GI(J.) log 2004	3839694
AR(2)+S.D.+UR+IC+GI(J.&F.S.) log 2004	1565497	AR(7)+S.D.+UR+IC+GI(J.&F.S.) log 2004	3861489
AR(6)+S.D.+UR+IC+GI(J.&F.S.) log 2004	1576811	AR(4)+S.D.+UR+IC+GI(J.&F.S.) log 2004	3887615
AR(6)+S.D.+UR+IC+GI(J.) log 2004	1593775	AR(6)+S.D.+UR+IC+GI(J.) log 2004	3914935
AR(7)+S.D.+UR+IC+GI(J.&F.S.) log 2004	1595086	AR(3)+S.D.+UR+IC+GI(J.&F.S.) log 2004	3939222
AR(3)+S.D.+UR+IC+GI(J.&F.S.) log 2004	1595117	AR(7)+S.D.+UR+IC+GI(J.) log 2004	3973551
AR(8)+S.D.+UR+IC+GI(J.&F.S.) log 2004	1608689	AR(8)+S.D.+UR+IC+GI(J.&F.S.) log 2004	3999943

In each row, the following information is reported: the model, the number of lags, (eventual) exogenous regressors, the data transformation, the first year of the estimation sample.

In general, Google-based models performed very well both for nowcasting and forecasting. In this regard, Table 7 shows that the number of models with Google data in the Top 100 ranked models in terms of RMSE is very high, particularly for nowcasting 2 steps ahead and forecasting, where more than 90 models include Google data.

In case of *nowcasting*, linear AR and ARMA models augmented with seasonal dummies were sufficient to provide good nowcasts of the food stamps data. Particularly, simple linear models using the log-returns of food stamps and no additional regressors, were sufficient for nowcasting 1 step ahead. Instead, ARX(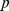) models with seasonal dummies and Google data were the best choice for nowcasting 2 steps ahead, see Table 9. Interestingly, the specification of the best models in this case is quite close to the one selected by the structural relationship identification methodology in Table 4. Moreover, the best models were those with the food stamps data in levels or in log-levels, thus confirming the previous evidence of stationarity.

As for *forecasting*, the evidence is strongly in favor of Google-based models, where all top models in terms of RMSE have a specification very close to the ARX(4) model with seasonal dummies reported in Table 4. Considering that we compared almost 3000 models, this is rather encouraging and confirms that the structural relationship identification methodology by [29] and [30] is a rather robust method of model selection. With regard to nonlinear models, only AAR models showed good performances, while this was not the case for the other three nonlinear models that we considered in our analysis: SETAR models did not reach numerical convergence under all possible configurations; LSTAR were a little bit better, but they were computationally demanding and almost 15% of the considered configurations did not reach convergence. Moreover, most of them had a ranking position above the 1500th place in terms of RMSE. Neural Networks were rather quick to estimate but similarly to LSTAR models they did not fare well in terms of ranking positions: in case of nowcasting, the majority of them ranked higher than the 1500th place, while in case of forecasting most of them ranked above the 1000th position. As for periodic models, simple PAR(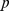) models and PECM models including only food stamps data and GIs performed rather very well in case of forecasting, whereas more complex configurations with periodic trends, ARCH effects or alternative periodic cointegration models performed rather poorly: most likely, the wealth of parameters that these complex periodic models involves resulted in very imprecise estimates. As for multivariate models, they were generally out of the top 100 models in terms of RMSE and most likely they suffered from efficient loss due to the high number of parameters (the only exception were Bayesian models which performed very good for nowcasting 1 step ahead).

We then tested for statistically significant differences in the forecast performances among the competing models by using the Model Confidence Set (MCS) approach developed by [71]. The MCS is a sequential test of equal predictive ability: given an initial set of forecasting models it tests the null that no forecasting model is distinguishable from any other, so that the starting hypothesis is that all models considered have equal forecasting performances. The MCS procedure yields a model confidence set containing the best forecasting models at some confidence level. An additional advantage of the MCS is that it acknowledges the limits to the informational content of the data: informative dataset will deliver a set that contains only the best model, while less informative data will not be able to distinguish between the competing models and the final set may contain several, if not all, models. We considered the maximum t statistic 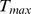, which is the default statistic in [71], as well as the semi-quadratic statistic 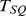, which is more computationally intensive but more selective (see e.g. [72] and [21] for some recent applications). The loss function used was the Mean Squared Error (MSE), while the p-values for the test statistic were obtained by using the stationary block bootstrap with a block length of 12 months and 1000 re-samples: if the p-value was lower than a defined threshold probability level 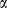, the model was not included in the MCS and vice-versa. We set 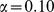 as in [71].

We report in Table 11 the number of models selected by the MCS procedure according to the MSE loss function, for nowcasting and forecasting. Moreover, we also report the number of selected models including Google data, as well as the number of selected nonlinear models.

**Table 11:** Number of models included in the MCS, at the 90% confidence level, using the 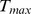 and 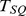 statistics and the MSE loss function.

	1 step		2 step		12 steps		24 step	
	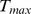	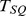	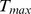	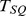	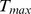	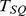	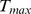	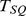
*Total n. of models selected*	683	6	119	2	11	87	37	20
*Google models*	334	2	102	2	11	79	37	20
*Nonlinear models*	7	0	0	0	0	3	0	0

In general, we can note that the number of models selected is quite small, with the only exception of the 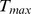 statistic for the case of nowcasting, which selected from a minimum of 119 models up to 683 models. In all other cases, the selected models are no more than 40, which indicates that our dataset is rather informative and it can be used to separate poor forecasting models from superior ones. Moreover, the semi-quadratic statistic is much more selective than the maximum t statistic, as expected, and in the case of nowcasting 2 steps ahead it selects only two models: the ARX(3) with seasonal dummies, data in log levels and all exogenous variables and the ARX(8) with seasonal dummies, data in levels and only one exogenous variables included (the GI for the keyword “food stamps”). With the exception of nowcasting 1 step ahead, models with Google data represent the vast majority of the models included in the MCS: this is quite strong evidence that Google online search data provide additional information content not included in standard economic variables.

The fact that simple linear models, augmented with the search volumes for the keywords “food stamps” and “jobs”, improve so much the forecasting at long horizons is an indirect confirmation of the recent analysis of food stamps caseload dynamics by [73] and [4], who showed that “*caseloads spell lengths had increased substantially from earlier time periods and… the impact of the current record caseloads may be felt for a long time to come*”, [[4], p. 327].

### Robustness checks

We wanted to verify that our promising results with Google data hold also with different assumptions, alternative forecasting environments and different comparison methodologies. Therefore, we performed the following robustness checks: a) we verified whether alternative keywords in Google Trends could be used for forecasting the number of food stamps recipients; b) we employed a recent falsification test proposed by [16]; c) we considered alternative out-of-sample intervals with and without the global financial crisis included; d) we compared the models in terms of directional accuracy; e) we repeated the same forecasting exercise for each of the 50 US states plus the Department of Columbia. All checks confirmed the superior forecasting performance of Google based models in general and of ARX models in particular, with specifications always close to that found using the structural relationship identification methodology by [29] and [30].

#### Alternative Keywords

**Figure 4:**
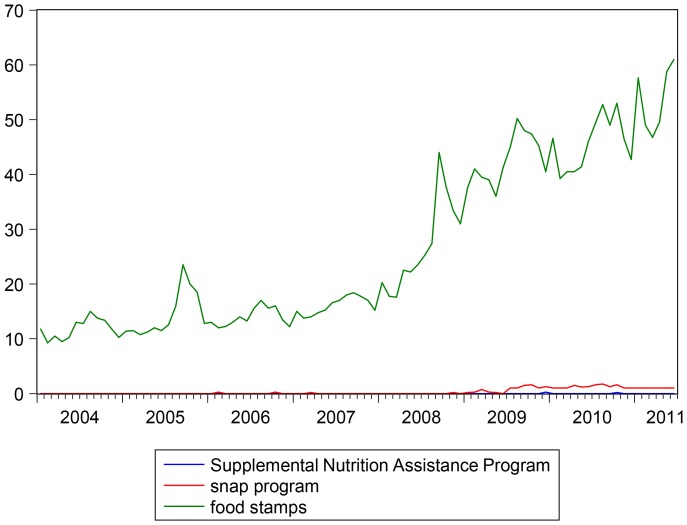
GIs for the keywords “*Supplemental Nutrition Assistance Program*”, “*snap program*”, and “*food stamps*”. Sample: 2004M1 - 2011M5. Search keywords are not case sensitive. Google data are registered trademarks of Google Inc., used with permission.

An important issue is to verify whether alternative keywords for Google searches can be used for forecasting purposes in the place of those used in the main analysis (i.e. “food stamps” and “jobs”). It is well known that in October 2008 the US farm bill renamed the Food Stamp Program as the *Supplemental Nutrition Assistance Program*. However, if we compare the online search volumes for this new name, together with “snap program” and the standard search “food stamps”, we can see that the keywords of interest remain only “food stamps” (see Figure 4): the alternative keywords *Supplemental Nutrition Assistance Program* and *snap program* have much lower search volumes and they start having Google indexes different from zero only from the end of 2008, so that they cannot be used in our forecasting exercise. Moreover, the vast majority of searches involving these alternative keywords also includes either “food stamps” or “food program”. Therefore, our case seems to be quite different from forecasting the US unemployment rate with Google data as in [16], where there can be alternative important keywords beside the main one given by “*jobs*”.

#### A Falsification Test using Google Correlate

Google has recently developed a new application called Google Correlate which can find out the web searches for keywords that either show the highest correlation with a given keyword search, or show the highest correlation with a given time series, given a specific time horizon. More specifically, its objective is to “*to surface the queries in the [Google] database whose spatial or temporal pattern is most highly correlated (* 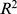 *) with a target pattern*” (official Google Correlate white paper, p. 2, available at http://www.google.com/trends/correlate/whitepaper.pdf). Google Correlate is basically Google Trends in reverse.

As a further check, we therefore decided to employ the falsification test proposed by [16], which considers the forecasting performance of an alternative GI chosen by Google Correlate: in our case, the keyword search that had the highest correlation with the US food stamps data during the in-sample period (2004M1-2007M2) was “*pci express slot*.” It is clear that such terms have nothing to do with the food stamps program. We thus added 375 forecasting models using the new Google Index to our previous 2890 models considered in the baseline case.

We do not report the top 10 models in terms of the Root Mean Square Error (RMSE) as in the previous section, since no single model using the “false” Google keywords was among the Top 100 models for every forecasting horizon. Instead, we report in Table 12 the number of models selected by the MCS procedure, together with the number of selected models using the “false” Google Index.

**Table 12:** Number of nowcasting and forecasting models selected in the MCS at the 90% confidence level, using the 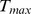 statistic and the MSE loss function, as well as number of selected models using the “false” Google Index.

	N. 1 step	N. 2 steps	F. 12 steps	F. 24 steps
Models selected	614	122	37	36
Models using the “false” Google Index	29	2	0	0

Table 12 shows that only a very limited number of models using the “false” GI were selected in case of nowcasting, while not a single model was selected in case of forecasting. These poor results were expected since the “false” Google data were completely disconnected from the food stamps program. Therefore, this evidence suggests that purely automatic methods (like Google Correlate) may not always represent the optimal keyword selection choice, see also [21] for similar results in case of oil data.

#### Different Out-Of-Sample Periods

We wanted to verify the forecasting performance of our competing models using different out-of-sample periods, to check the robustness of our results to different business cycle conditions: for example, our preliminary in-sample analysis highlighted a potential structural break for Google based models in 2008, with a timing close to the Lehman Brothers bankruptcy. In this regard, we followed the approach suggested by [16] and considered the following two alternative out-of-samples:

- 2008M10-2011M5: this sample starts just after the bankruptcy of Lehman Brothers;
- 2009M7- 2011M5: this second sample starts with the end of the (official) NBER recession for the US in 2009.

Due to the new out-of-samples dimensionality, forecasts 24 steps ahead are considered only for the second sample starting in 2009. The top 10 models in terms of the Root Mean Square Error (RMSE) for nowcasting and forecasting are reported in Tables 13–16, together with the top 10 models for the baseline case, which are reported in the left column for ease of reference; Table 17 reports the number of models selected by the MCS procedure according to the MSE loss function and the 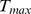 statistic at the 90% confidence level, together with the number of selected models using Google data.

**Table 13:** Top 10 models in terms of RMSE - different out-of-sample periods. Nowcasting: 1 step ahead.

**1 STEP ahead (baseline case)**	**1 STEP ahead (recession 2008)**
**Top 10 models**	**Top 10 models**
ARMA(10,10) dlog 1988	BVAR(1,12) FS+UR+IC+GI(F.S.) lev 2004
ARMA(10,10) + UR dlog 1988	BVAR(7) FS+UR+IC+GI(F.S.) lev 2004
ARMA(12,12) dlog 1988	AR(4)+S.D.+GI(F.S.) lev 2004
ARMA(11,11)+UR diff 1988	AR(7)+S.D.+GI(F.S.) lev 2004
AR(12) + IC (sim + lags) dlog 1988	AR(5)+S.D.+GI(F.S.) lev 2004
ARMA(12,12) + IC diff 1988	AR(8)+S.D.+GI(F.S.) lev 2004
BVAR(1,12) FS+GI(F.S.) lev 2004	AR(6)+S.D.+GI(F.S.) lev 2004
BVAR(1,12) FS+UR+IC+GI(F.S.) lev 2004	ARMA(11,11)+UR diff 1988
ARMA(12,12) + UR dlog 1988	ARMA(12,12)+GI(J.&F.S.) lev 2004
ARMA(11,11) dlog 1988	AR(6)+S.D.+GI(J.&F.S.) log 2004
**1 STEP ahead (baseline case)**	**1 STEP ahead (expansion 2009)**
**Top 10 models**	**Top 10 models**
ARMA(10,10) dlog 1988	AR(8)+S.D.+IC lev 2004
ARMA(10,10) + UR dlog 1988	AR(9)+S.D.+IC lev 2004
ARMA(12,12) dlog 1988	AR(7)+S.D.+IC lev 2004
ARMA(11,11) + UR diff 1988	AR(7)+S.D.+UR+IC+GI(J.) log 2004
AR(12) + IC (sim + lags) dlog 1988	AR(10)+S.D.+IC lev 2004
ARMA(12,12) + IC diff 1988	AR(8)+S.D.+UR+IC+GI(J.) log 2004
BVAR(1,12) FS+GI(F.S.) lev 2004	AR(6)+S.D.+GI(J.) log 2004
BVAR(1,12) FS+UR+IC+GI(F.S.) lev 2004	AR(4)+S.D.+IC lev 2004
ARMA(12,12) + UR dlog 1988	AR(5)+S.D.+IC lev 2004
ARMA(11,11) dlog 1988	AR(10)+S.D.+UR+IC+GI(J.) log 2004

Baseline case (left column) and the two cases including the 2008 recession (top right column) and the expansion starting in 2009 (low right column).

**Table 14:** Top 10 models in terms of RMSE - different out-of-sample periods. Nowcasting: 2 steps ahead.

**2 STEPS ahead (baseline case)**	**2 STEPS ahead (recession 2008)**
**Top 10 models**	**Top 10 models**
AR(3)+S.D.+UR+IC+GI(J.&F.S.) log 2004	AR(8)+S.D.+GI(F.S.) lev 2004
AR(8)+S.D.+GI(F.S.) lev 2004	AR(7)+ S.D.+GI(F.S.) lev 2004
AR(7)+ S.D.+GI(F.S.) lev 2004	AR(10)+ S.D.+GI(F.S.) lev 2004
AR(2)+S.D.+UR+IC+GI(J.&F.S.) log 2004	AR(4)+ S.D.+GI(F.S.) lev 2004
AR(7)+S.D.+GI(J.&F.S.) lev 2004	AR(9)+ S.D.+GI(F.S.) lev 2005
AR(4)+ S.D.+GI(F.S.) lev 2004	AR(5)+ S.D.+GI(F.S.) lev 2006
AR(4)+S.D.+GI(J.&F.S.) lev 2004	AR(6)+ S.D.+GI(F.S.) lev 2007
AR(8)+S.D.+GI(J.&F.S.) lev 2004	AR(11)+ S.D.+GI(F.S.) lev 2007
AR(5)+ S.D.+GI(F.S.) lev 2004	ARMA(12,12)+GI(J.&F.S.) log 2004
AR(6)+S.D.+UR+IC+GI(J.&F.S.) log 2004	AR(6)+S.D.+GI(J.&F.S.) log 2004
**2 STEPS ahead (baseline case)**	**2 STEPS ahead (expansion 2008)**
**Top 10 models**	**Top 10 models**
AR(3)+S.D.+UR+IC+GI(J.&F.S.) log 2004	AR(8)+S.D.+IC lev 2004
AR(8)+S.D.+GI(F.S.) lev 2004	AR(7)+S.D.+IC lev 2004
AR(7)+ S.D.+GI(F.S.) lev 2004	AR(10)+S.D.+IC lev 2004
AR(2)+S.D.+UR+IC+GI(J.&F.S.) log 2004	AR(7)+S.D.+UR+IC+GI(J.&F.S.) log 2004
AR(7)+S.D.+GI(J.&F.S.) lev 2004	AR(9)+S.D.+IC lev 2004
AR(4)+ S.D.+GI(F.S.) lev 2004	AR(2)+S.D.+UR+IC+GI(F.S.) lev 2004
AR(4)+S.D.+GI(J.&F.S.) lev 2004	AR(2)+S.D.+UR+IC+GI(J.&F.S.) log 2004
AR(8)+S.D.+GI(J.&F.S.) lev 2004	AR(6)+S.D.+UR+IC+GI(J.&F.S.) log 2004
AR(5)+ S.D.+GI(F.S.) lev 2004	AR(3)+S.D.+UR+IC+GI(F.S.) log 2004
AR(6)+S.D.+UR+IC+GI(J.&F.S.) log 2004	AR(5)+S.D.+IC lev 2004

Baseline case (left column) and the two cases including the 2008 recession (top right column) and the expansion starting in 2009 (low right column).

**Table 15:** Top 10 models in terms of RMSE - different out-of-sample periods. Forecasting: 12 step ahead.

**12 STEPS ahead (baseline case)**	**12 STEPS ahead (recession 2008)**
**Top 10 models**	**Top 10 models**
AR(2)+S.D.+UR+IC+GI(J.) log 2004	PAR(1)+UR+IC+GI(J.) log 2004
AR(5)+S.D.+UR+IC+GI(J.&F.S.) log 2004	AR(2)+S.D.+UR+IC+GI(J.) log 2004
AR(3)+S.D.+UR+IC+GI(J.) log 2004	AR(3)+S.D.+UR+IC+GI(J.) log 2004
AR(4)+S.D.+UR+IC+GI(J.&F.S.) log 2004	PAR(1)+UR+IC+GI(J.) lev 2004
AR(2)+S.D.+UR+IC+GI(J.&F.S.) log 2004	PAR(1)+UR+IC+GI(J.&F.S.) lev 2004
AR(6)+S.D.+UR+IC+GI(J.&F.S.) log 2004	PAR(1)+UR+IC+GI(J.&F.S.) log 2004
AR(6)+S.D.+UR+IC+GI(J.) log 2004	AR(5)+S.D.+UR+IC+GI(J.&F.S.) log 2004
AR(7)+S.D.+UR+IC+GI(J.&F.S.) log 2004	AR(6)+S.D.+UR+IC+GI(J.) log 2004
AR(3)+S.D.+UR+IC+GI(J.&F.S.) log 2004	AR(4)+S.D.+UR+IC+GI(J.&F.S.) log 2004
AR(8)+S.D.+UR+IC+GI(J.&F.S.) log 2004	AR(5)+S.D.+UR+IC+GI(J.) log 2004
**12 STEPS ahead (baseline case)**	**12 STEPS ahead (expansion 2009)**
**Top 10 models**	**Top 10 models**
AR(2)+S.D.+UR+IC+GI(J.) log 2004	PAR(1)+UR+IC+GI(J.&F.S.) log 2004
AR(5)+S.D.+UR+IC+GI(J.&F.S.) log 2004	AR(4)+S.D.+GI(J.&F.S.) log 2004
AR(3)+S.D.+UR+IC+GI(J.) log 2004	AR(5)+S.D.+GI(J.&F.S.) log 2004
AR(4)+S.D.+UR+IC+GI(J.&F.S.) log 2004	AR(6)+S.D.+GI(J.&F.S.) log 2004
AR(2)+S.D.+UR+IC+GI(J.&F.S.) log 2004	PAR(1)+UR+IC+GI(J.&F.S.) lev 2004
AR(6)+S.D.+UR+IC+GI(J.&F.S.) log 2004	AR(7)+S.D.+GI(J.&F.S.) log 2004
AR(6)+S.D.+UR+IC+GI(J.) log 2004	AR(4)+S.D.+UR+IC+GI(J.&F.S.) log 2004
AR(7)+S.D.+UR+IC+GI(J.&F.S.) log 2004	AR(5)+S.D.+UR+IC+GI(J.&F.S.) log 2004
AR(3)+S.D.+UR+IC+GI(J.&F.S.) log 2004	PAR(1)+UR+IC+GI(J.) log 2004
AR(8)+S.D.+UR+IC+GI(J.&F.S.) log 2004	AR(2)+S.D.+UR+IC+GI(J.) log 2004

Baseline case (left column) and the two cases including the 2008 recession (top right column) and the expansion starting in 2009 (low right column).

**Table 16:** Top 10 models in terms of RMSE - different out-of-sample periods. Forecasting: 24 steps ahead.

24 STEPS ahead (baseline case)	24 STEPS ahead (expansion 2009)
Top 10 models	Top 10 models
AR(6)+S.D.+UR+IC+GI(J.&F.S.) log 2004	AR(2)+S.D.+UR+IC+GI(J.) log 2004
AR(5)+S.D.+UR+IC+GI(J.&F.S.) log 2004	AR(6)+S.D.+UR+IC+GI(J.) log 2004
AR(2)+S.D.+UR+IC+GI(J.&F.S.) log 2004	AR(3)+S.D.+UR+IC+GI(J.) log 2004
AR(2)+S.D.+UR+IC+GI(J.) log 2004	AR(5)+S.D.+UR+IC+GI(J.) log 2004
AR(7)+S.D.+UR+IC+GI(J.&F.S.) log 2004	AR(7)+S.D.+UR+IC+GI(J.) log 2004
AR(4)+S.D.+UR+IC+GI(J.&F.S.) log 2004	AR(8)+S.D.+UR+IC+GI(J.) log 2004
AR(6)+S.D.+UR+IC+GI(J.) log 2004	AR(9)+S.D.+UR+IC+GI(J.) log 2004
AR(3)+S.D.+UR+IC+GI(J.&F.S.) log 2004	AR(11)+S.D.+UR+IC+GI(J.) log 2004
AR(7)+S.D.+UR+IC+GI(J.) log 2004	AR(4)+S.D.+UR+IC+GI(J.) log 2004
AR(8)+S.D.+UR+IC+GI(J.&F.S.) log 2004	AR(6)+S.D.+UR+IC+GI(J.&F.S.) log 2004

Baseline case (left column) and the case including the expansion starting in 2009 (low right column).

**Table 17:** Number of nowcasting and forecasting models selected in the MCS at the 90% confidence level, using the 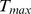 statistic and the MSE loss function, as well as number of selected Google based models.

		Recession 2008	Expansion 2009
Nowcasting 1 step	Models selected	173	82
	Google based models	101	68
Nowcasting 2 steps	Models selected	101	51
	Google based models	89	42
Forecasting 12 steps	Models selected	22	5
	Google based models	22	5
Forecasting 24 steps	Models selected	NA	13
	Google based models	NA	13

Tables 13–14 show that, in case of nowcasting, Google based models tend to do particularly well during the recession period, while models using the unemployment rate and initial claims tend to perform better in terms of RMSE during the economic expansion, even though in the latter case the difference is rather small. This evidence is confirmed by the Model Confident Set approach, where more than 60% of the selected models are Google based models, for both out-of-sample periods. As for forecasting 12 and 24 steps ahead, Tables 15–16 show that the results are quite similar to the baseline case instead, with Google based models in the top spots. Moreover, all selected models by the MCS are Google based models. Interestingly, these two alternative out-of-sample periods are much more informative for the MCS approach, because the number of selected models is much lower compared to the baseline case reported in Table 11 (particularly for nowcasting). Therefore, this evidence highlights that Google models are much more stable than competing models, and their forecasting performances are robust across different business cycles, as recently found also by [16] and [21].

#### Directional Accuracy and Turning Points: Can Google Help

The analysis has considered so far only the accuracy of forecasts in terms of magnitude, but also directional accuracy is important: even if forecast errors are large, forecasts with the correct direction of change may still provide useful information about food stamps dynamics. A special case of directional accuracy is the ability to predict a turning point, which is a change in the direction of movement of the variable under investigation, and it exists if 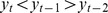 (peak turning point) or 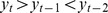 (trough turning point), see [74] and [75] for details.

Unfortunately, our forecasting evaluation period (2007M3-2011M5) spans a limited time sample, where food stamps caseloads mainly increased (see Figure 2). Nevertheless, if we evaluate the directional accuracy of the competing forecasting models, we are still able to identify a limited group of best models, at least for nowcasting (see Table 18).

**Table 18:** Directional accuracy of forecasts: number of models with 100% correct predictions for the direction of change.

	N. 1 step	N. 2 steps	F. 12 steps	F. 24 steps
N. of models	1	179	1096	1252
Google based models	1	101	715	815

Table 18 shows that there was only 1 model able to correctly predict all the 51 directions of change in case of nowcasting 1 step ahead, and this is a Google based model (the AR(3)+UR+IC+GI(“food stamps”) model using log-transformed data). Instead, it is not a surprise that the number of models with a 100% directional accuracy increases with the forecast horizon, since the number of food stamps was continuously increasing in the considered forecasting sample: therefore, directional accuracy cannot be used to discriminate competing models in this case.

**Figure 5:**
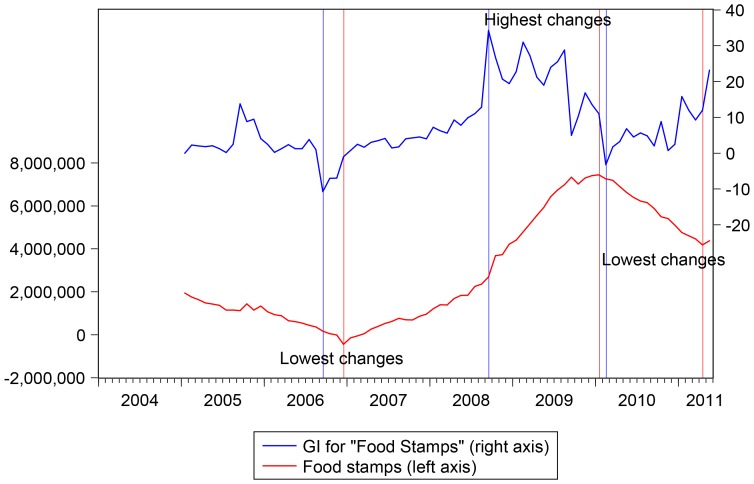
Yearly changes for the food stamps data and the GI for “food stamps”. Sample: 2004M1 - 2011M5. The turning points for each series is highlighted by a vertical line of the same color. Google data are registered trademarks of Google Inc., used with permission.

As for turning points, we could not evaluate the models' ability to predict them because there were none in the forecasting sample. Nevertheless, a simple indirect way to check whether the forecasting models would have been able to predict them is to evaluate their ability to correctly forecast the sign of second order differenced data (that is 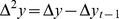). To get an intuitive idea of this point, we plot in Figure 5 the yearly changes 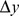 of the number of food stamps recipients and the Google Index for “food stamps”: even though the yearly changes of food stamps data were almost always positive, they had a declining rate between 2004 and 2006 (that is 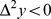), an increasing rate between 2006 and 2009 (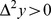) and again a declining rate between 2010 and 2011 (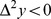). Interestingly, the yearly changes of the GI for the keywords “food stamps” showed a similar pattern, which always anticipated the turning points of the yearly changes of food stamps data: from a minimum of 3 months in advance in 2006, up to 16 months in 2008 and 14 months in 2010. Therefore, Figure 5 gives some clues for understanding why Google based models forecasted so well food stamps 12 steps and 24 steps ahead.

The directional accuracy of the competing models for the second order differenced data 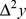 is reported in Table 19. Given that the selection is now much higher than for the first order changes discussed in Table 18, we report both the first-best models and the second-best models.

**Table 19:** Directional accuracy for 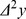: number of first-best and second-best models, together with their percentage of correct predictions for the sign of 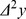.

		N. 1 step		N. 2 steps		F. 12 steps		F. 24 steps	
		*N.*	*% correct*	*N.*	*% correct*	*N.*	*% correct*	*N.*	*% correct*
(1st best)	Models total	6	82%	3	80%	1	79%	2	81%
	Google based models	1	82%	0	/	0	/	1	81%
(2nd best)	Models total	12	80%	7	78%	2	77%	6	78%
	Google based models	1	80%	4	78%	0	/	5	78%

The results in Table 19 are somewhat mixed but partially confirm what we previously found out when examining the accuracy of forecasts in terms of magnitude: simple linear models augmented with initial claims and the unemployment rate are sufficient for nowcasting food stamps, while Google based models perform better for nowcasting 2 steps ahead and for long term forecasting (24 steps ahead).

#### US State Level Forecasts

The last check was to estimate the same set of forecasting models for each of the 50 US states, together with the District of Columbia. A similar check was implemented by [16] when forecasting the US unemployment rate with Google data. As in the baseline case, the evaluation period ranged from 2007M3 till 2011M5 and was used to compare the nowcasts 1 step and 2 steps ahead, as well as the forecasts 12 steps and 24 steps ahead. For sake of interest and space, we report in Table 20 the number of models using Google data among the Top 100 models according to the RMSE for each US state. Moreover, the same table also reports the US census state population and the population density per square mile as of April 1, 2010.

**Table 20:** US state level forecasts.

US state	1 step	2 steps	12 steps	24 steps	Census	Density (inhabitants
					Population	per square mile)
Alabama	38	100	89	87	4822023	92
Alaska	45	61	62	38	731449	1
Arizona	44	36	4	20	6392017	56
Arkansas	0	9	0	26	2915918	55
California	34	68	84	72	37253956	228
Colorado	1	3	4	53	5029196	48
Connecticut	57	48	17	94	3574097	645
Delaware	24	52	42	40	897934	361
District of Columbia	2	1	41	14	601723	8805
Florida	0	13	13	42	18801310	286
Georgia	35	35	58	75	9687653	163
Hawaii	20	30	35	35	1360301	124
Idaho	35	36	11	10	1567582	19
Illinois	38	68	87	67	12830632	222
Indiana	30	48	22	8	6483802	178
Iowa	34	60	38	23	3046355	54
Kansas	100	98	18	51	2853118	35
Kentucky	66	64	41	44	4339367	107
Louisiana	28	14	98	94	4533372	87
Maine	21	24	51	57	1328361	38
Maryland	64	70	43	60	5296486	427
Massachusetts	73	65	63	50	6349097	602
Michigan	64	65	62	66	9938444	103
Minnesota	16	8	45	65	4919479	57
Mississippi	7	3	35	39	2844658	59
Missouri	8	0	2	1	5595211	80
Montana	53	48	35	36	902195	6
Nebraska	1	0	9	95	1711263	22
Nevada	39	26	13	21	1998257	18
New Hampshire	60	14	41	86	1235786	132
New Jersey	57	72	84	87	8414350	965
New Mexico	43	43	46	58	1819046	15
New York	1	14	76	72	18976457	348
North Carolina	65	80	83	74	8049313	150
North Dakota	4	17	41	38	642200	9
Ohio	16	18	60	73	11353140	253
Oklahoma	57	65	6	14	3450654	49
Oregon	74	56	24	2	3421399	35
Pennsylvania	78	76	49	67	12281054	267
Rhode Island	28	46	76	93	1048319	679
South Carolina	65	70	41	42	4012012	125
South Dakota	43	55	53	95	754844	10
Tennessee	6	53	46	33	5689283	135
Texas	16	75	70	60	20851820	78
Utah	39	56	31	39	2233169	26
Vermont	18	33	29	20	608827	63
Virginia	73	74	69	40	7078515	165
Washington	47	44	29	49	5894121	83
West Virginia	1	12	23	24	1808344	75
Wisconsin	12	4	4	15	5363675	82
Wyoming	5	8	52	33	493782	5

Number of models using Google data out of the Top 100 models (according to the RMSE), 2010 census population data for each US state and population density (inhabitants per square mile, 2010).

The results are quite similar to what we saw for the whole US (not surprisingly). However, two outcomes are worth noticing: Google data seems to be more useful for forecasting highly densely populated US states, while its importance is minor for several states with small population and low density. Probably, this may be due to a higher internet penetration in highly densely populated states. However, this issue goes beyond the scope of this paper and we leave it as an interesting avenue for further research. Secondly, the number of models with Google data in the Top 100 increases with the forecasting horizon, thus confirming similar evidence in [16].

## Conclusion

We proposed the use of Google data based on internet searches about food stamps as a potential indicator to nowcast and forecast the US monthly number of individuals participating in the Supplemental Nutrition Assistance Program, formerly known as the Food Stamp program. We compared almost 3000 forecasting models and we found that Google based models definitively improved nowcasting food stamps 2 months ahead, while simple linear models (eventually augmented with unemployment rates or initial claims data) are sufficient for nowcasting 1 month ahead. Moreover, Google based models provided statistically significant superior forecasts in case of forecasting 12 steps and 24 steps ahead. More specifically, linear autoregressive models augmented with Google search data for the terms “food stamps” and “jobs”, the unemployment rate and initial claims were the best models for forecasting purposes. In this regard, the best models had specifications always close to the ARX(4) model found using the structural relationship identification methodology by [29] and [30] in the in-sample analysis, thus showing that this approach is a rather robust method of model selection in case of small samples.

Nonlinear models performed poorly, were computationally intensive, and in several cases did not reach numerical convergence, with the exception of additive autoregressive models which provided competitive forecasts in case of long term forecasting. Simple periodic autoregressive models performed quite well for 12-step and 24-step ahead forecasts, while more complex periodic models performed poorly, probably due to the high number of estimated parameters which hindered their forecasting performances. Our results hold also with alternative out-of-sample periods which either include the global financial crisis or start after the (official) end of this recession. Besides, our Google based models passed a falsification test which considered the forecasting performance of an alternative Google index chosen by Google Correlate. Similar results were also found when considering the directional accuracy of the models' forecasts. Finally, the estimates for single US states gave similar results to the case of the whole US, even though we found that Google data are very important in case of highly densely populated US states, while their importance is minor for several states with small population.

We remark that although we considered a very large set of models, we had to restrict the potential range of models to keep the forecasting exercise computationally tractable. An avenue of future research would be to consider additional models like fractional cointegration, exponential smoothing methods in state space form and many others.

## Supporting Information

Software Description S1 **The Software description.**
(PDF)

Data S1 **The (cleaned) food stamps data.**
(ZIP)
