# Supplementary material for: Nowcasting and Forecasting the Monthly Food Stamps Data in the US Using Online Search Data
Source: PLoS One. 2014 Nov 4;9(11):e111894. doi: 10.1371/journal.pone.0111894 (PMC4219814; doi:10.1371/journal.pone.0111894)
Supplement: Software Description S1 — The Software description. (PDF) [file pone.0111894.s001.pdf]

"Nowcasting and Forecasting the Monthly Food Stamps Data in the US using Online Search Data"

Supporting Information

SOFTWARE

Unit Root Tests:

- ***Non periodic unit root tests with breaks***:
  - Lee and Strazicich (2003) unit root test allowing for 1 or 2 structural breaks: the original Gauss code can be downloaded from Prof. Junsoo Lee's website,
    http://old.cba.ua.edu/∼jlee/gauss
  - The Range Unit Root test and the Forward-Backward RUR test by Aparicio, Escribano and Garcia (2006) can be programmed in every statistical/econometric packages. The R code written by the author will be posted on the author's personal website.

- ***Periodic unit root tests***: the periodic unit root tests based on PAR models can be easily programmed in every statistical/econometric packages. Some `Eviews` code is reported in the Appendix of the book by Franses and Paap (2004).

Weak Exogeneity tests::

- The Wald test for weak exogeneity is implemented in almost all econometric and statistical software available, whereas the Wald test by Toda and Yamamoto (1995) is a straightforward extension of the standard Wald test. A step-by-step example using Eviews by prof. David Giles can be found here:

  http://davegiles.blogspot.com/2011/04/testing-for-granger-causality.html

Cointegration tests::

- ***Non periodic cointegration tests without structural break(s)***: the Engle and Granger (1987) single-equation test and Johansen (1995) multivariate test are implemented in almost all econometric and statistical software available, so that it is only a matter of the researcher' preferences which one he/she selects.

- ***Non periodic cointegration tests with structural break(s)***:
  - Gregory and Hansen (1996) single-equation test with 1 endogenous break: the original `Gauss/R/Matlab` code can be downloaded from Prof. Bruce Hansen's website,
    http://www.ssc.wisc.edu/∼bhansen/progs/joe_96.html
    An `Eviews` routine for the Gregory-Hansen cointegration test with 1 endogenous structural break can also be found here:
    http://forums.eviews.com/viewtopic.php?f=15&t=976&start=15
    A `Stata` module for Gregory-Hansen cointegration test with 1 endogenous structural break can be found at Repec:
    http://ideas.repec.org/c/boc/bocode/s457327.html
  - Hatemi (2008) single-equation test with 2 endogenous breaks: the original `Gauss` code by Prof. Hatemi can be downloaded from Repec,
    http://econpapers.repec.org/software/bocbocode/g00006.htm
  - Johansen et al. (2000) multivariate test allowing up to two exogenous structural breaks either in levels or in levels and trend jointly: this test is implemented in `Jmulti`, www.jmulti.de which is a Java program interfaced withGauss programs. Moreover, `R` and `Eviews` programs to compute p-values and critical values for this test can be found here:
    http://web.uvic.ca/∼dgiles/downloads/johansen/index.html

Stability tests for linear models and optimal number of breakpoints: these tests are implemented in the `R` packages `strucchange` and `fxregime`. The optimal number of breakpoints can be computed with the same packages as well as with `Eviews 8`, where several (but not all) stability tests can also be found.

Misspecification tests for linear models: available in `Eviews` 6 or higher, as well as in several other softwares.

Estimation of linear models (AR, ARMA): these models are implemented in almost all econometric and statistical software available, so that it is only a matter of the researcher' preferences which one he/she selects.

Estimation of periodic models: a lot of Eviews code is reported in Franses and Paap (2004), which can be easily modified to produce several model extensions.

Estimation of VEC models: they are implemented in almost all econometric and statistical software available, so that it is only a matter of the researcher' preferences which one he/she selects.

Estimation of PEC models: they can be easily programmed in several statistical/econometric softwares. Some Eviews code is reported in Franses and Paap (2004).

Estimation of Nonlinear models (SETAR, LSTAR, AAR, NNET): these models are estimated using the `tsDyn` package in `R`, which is interfaced with the `mgcv` package for `AAR` models and with the `nnet` package for neural networks.

Model Confidence Set (MCS): this approach is implemented in the `Ox` package `Mulcom` which can be found here:
http://mit.econ.au.dk/vip_htm/alunde/mulcom/mulcom.htm
This package can be run with the `Ox` console which is free for academic research, study and teaching purposes and can be downloaded at http://www.doornik.com/download.html .
